# Beyond FoxP3—Identification of a Chicken Regulatory T Cell Signature

**DOI:** 10.1002/eji.70106

**Published:** 2025-12-19

**Authors:** Isabell Naumann, Edward S. Ricemeyer, Daniel Elleder, Jiri Plachy, Dominik von La Roche, Kim Vučinić, Thomas W. Göbel, Bernd Kaspers, Simon P. Früh, Sonja Härtle

**Affiliations:** ^1^ Department of Veterinary Sciences, AG Immunology Ludwig‐Maximilians‐Universität München Munich Germany; ^2^ Bond Life Sciences Center University of Missouri Columbia Missouri USA; ^3^ Palaeogenomics Group, Institute of Palaeoanatomy, Domestication Research and the History of Veterinary Medicine Ludwig‐Maximilians‐Universität Munich Germany; ^4^ Laboratory of Viral and Cellular Genetics Institute of Molecular Genetics of the Czech Academy of Sciences Prague Czech Republic; ^5^ Laboratory of Genomics and Bioinformatics Institute of Molecular Genetics of the Czech Academy of Sciences Prague Czech Republic; ^6^ Department of Veterinary Medicine Institute of Virology Freie Universität Berlin Berlin Germany

**Keywords:** chicken, CTLA‐4, FoxP3, single‐cell RNA‐seq, Treg signature

## Abstract

Regulatory T cells (Tregs), defined by the lineage‐specific transcription factor FoxP3, are crucial for immune regulation and have been studied extensively in mammals. However, avian Tregs remain poorly characterized, leaving gaps in our understanding of their evolutionary conservation and unique features. In this study, we investigated the phenotype of chicken Tregs to define reliable markers for their identification and characterization. We analyzed CD4^+^ splenocytes sorted into CD25^negative^, CD25^low^, and CD25^high^ subpopulations using RNA sequencing. *FOXP3* and other Treg‐associated genes were expressed in both CD25^low^ and CD25^high^ populations, showing that CD25 expression alone is insufficient to distinguish chicken Tregs. To refine the marker profile, we evaluated additional markers, including CTLA‐4 and GITR. Notably, we describe for the first time a chicken‐specific CTLA‐4 antibody, which uniquely stains CTLA‐4 exclusively in intracellular (ic) compartments, distinguishing it from mammalian counterparts. Single‐cell RNA sequencing further confirmed distinct *FOXP3* ^+^ clusters enriched for expression of *CTLA4* and *TNFRSF18* (encoding GITR). While CTLA‐4's ic expression limits usability in functional assays, the combination of CD4^+^/CD25^+^/CTLA‐4^+^/GITR^+^ represents the most accurate characterization of putative chicken Tregs to date. These findings highlight evolutionary conservation and species‐specific differences in Treg markers, providing the foundation for future studies on chicken Treg functionality.

## Graphical abstract

Chicken *FOXP3*+ putative regulatory T cells were identified using both bulk and single‐cell RNA sequencing to define their transcriptional signature and flow cytometry to confirm surface marker expression. This combined approach revealed a distinct putative Treg subset characterized by CD4, CD25, and GITR surface expression.

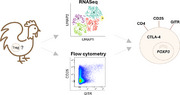

**Abbreviations**
IBDV: infectious bursal disease virus
ic: intracellular
iTreg: induced Treg
LFC: log_2_ fold change
MDV: Marek's disease virus
nTreg: natural Treg
RT‐qPCR: reverse transcription quantitative polymerase chain reaction
scRNA‐seq: single cell RNA sequencing
Treg: regulatory T cell

## 1 Introduction

T helper cells constitute a highly diverse set of lymphocytes with distinct phenotypical and functional properties. Among these cells, regulatory T cells (Tregs) play a unique role in immune tolerance, maintaining tissue homeostasis and limiting inflammation [1, 2]. They were first described in mice as CD4^+^ lymphocytes expressing high levels of the IL‐2 receptor alpha chain (CD25) [3] and the canonical transcription factor FoxP3. Based on their origin, CD4^+^ CD25^+^ FoxP3^+^ Tregs are classified into two main subtypes: thymus‐derived “natural” Tregs (nTregs) [4] and peripherally induced Tregs (iTregs) generated from naive CD4^+^ CD25^negative^ T cells via TGF‐β1 and IL‐2 [5].

In addition to CD4 and CD25, mammalian Tregs express characteristic molecules and surface markers for proper identification (Supporting information Table S1). Tregs modulate immune responses by cell contact‐dependent interactions via CTLA‐4, inhibitory cytokine secretion of IL‐10 and TGF‐β, and competition for growth factors [6]. By expressing the high‐affinity IL‐2 receptor alpha chain (CD25), Tregs deplete IL‐2, thus limiting effector cell proliferation and activity [6]. Despite their functional importance, Tregs constitute only 5%–10% of CD4^+^ T cells [7].

Although Tregs are well‐characterized in different mammalian species, knowledge about their evolution is limited. Identification of FoxP3 orthologues in fish [8], amphibians [9], and birds suggests evolutionary conservation of FoxP3 throughout vertebrates. However, functional data that could elucidate conserved immunoregulatory properties remain limited. One reason is that due to its intracellular localization, FoxP3 is not suitable for the isolation of viable cells, and defining additional Treg surface markers has proven challenging beyond human and mouse.

The chicken is a highly valuable immunological model. Birds and mammals separated more than 300 million years ago [10], and their immune systems share many similarities, but also exhibit distinct features, allowing for the identification of conserved and lineage‐specific immune mechanisms. Major differences in B cell development, such as the Bursa of Fabricius (bursa) as the primary B cell organ, were found in chickens. The avian T cell system shares similarities with mammalian T cells but also differs, for example, in the high prevalence of γδ T cells [11] and a differently structured MHC system. As chickens lack classical lymph nodes [11], there must also be alternative sites for B‐ and T‐cell interaction.

A few studies have identified T cells with regulatory functions in chickens [12] and ducks [13] within the CD4⁺ CD25⁺ subpopulation. Chicken CD4⁺ CD25⁺ T cells suppress responder T cell proliferation via contact‐dependent mechanisms that are partially reversible by IL‐2 [12]. In contrast, duck CD4⁺ CD25⁺ T cells can inhibit naïve T cell proliferation by a contact‐independent suppressive mechanism [13]. In addition, the recent identification of the chicken FoxP3 ortholog also provides strong evidence for the presence of Tregs in birds. Interestingly, *FOXP3* was detected in both CD25^low^ and CD25^high^ T helper cells [14]. Despite multiple attempts, generating monoclonal antibodies (mAbs) against chicken FoxP3 has proven challenging, and existing mammalian mAbs do not cross‐react, highlighting the need for additional Treg markers for flow cytometry.

Beyond FoxP3, the phenotypic characterization of avian Tregs remains limited. qPCR analysis indicated increased mRNA levels of the regulatory cytokines IL‐10 and TGF‐β in CD4⁺ CD25^+^ cells [12]. Furthermore, a specific subpopulation of CD4⁺ CD25⁺ lymphocytes that express TGF‐β on their surface has been described as putative chicken Tregs based on their involvement in Marek's disease virus (MDV) pathogenesis [15].

These findings suggest that there is likely a specific subpopulation of chicken CD4⁺ CD25⁺ cells exhibiting phenotypic and functional characteristics similar to those of mammalian Tregs; however, the precise phenotype of this subpopulation remains to be determined. Therefore, we used RNA sequencing of distinct CD25^negative^, CD25^low^, and CD25^high^ CD4⁺ T cell subpopulations, along with single‐cell sequencing of CD4⁺ T cells from chicken splenocytes, to better characterize *FOXP3*⁺ subsets. Transcriptome data were complemented with analysis of protein expression by establishing anti‐chicken CTLA‐4 antibodies and testing alongside a panel of potential Treg markers. This approach provides a detailed characterization of putative regulatory T cell subsets in chickens, offering valuable insights into their phenotype and diversity.

## 2 Results

### 2.1 Differences in Gene Expression in CD4^+^ CD25^negative^, CD4^+^ CD25^low^, and CD4^+^ CD25^high^ Subpopulations

**FIGURE 1:**
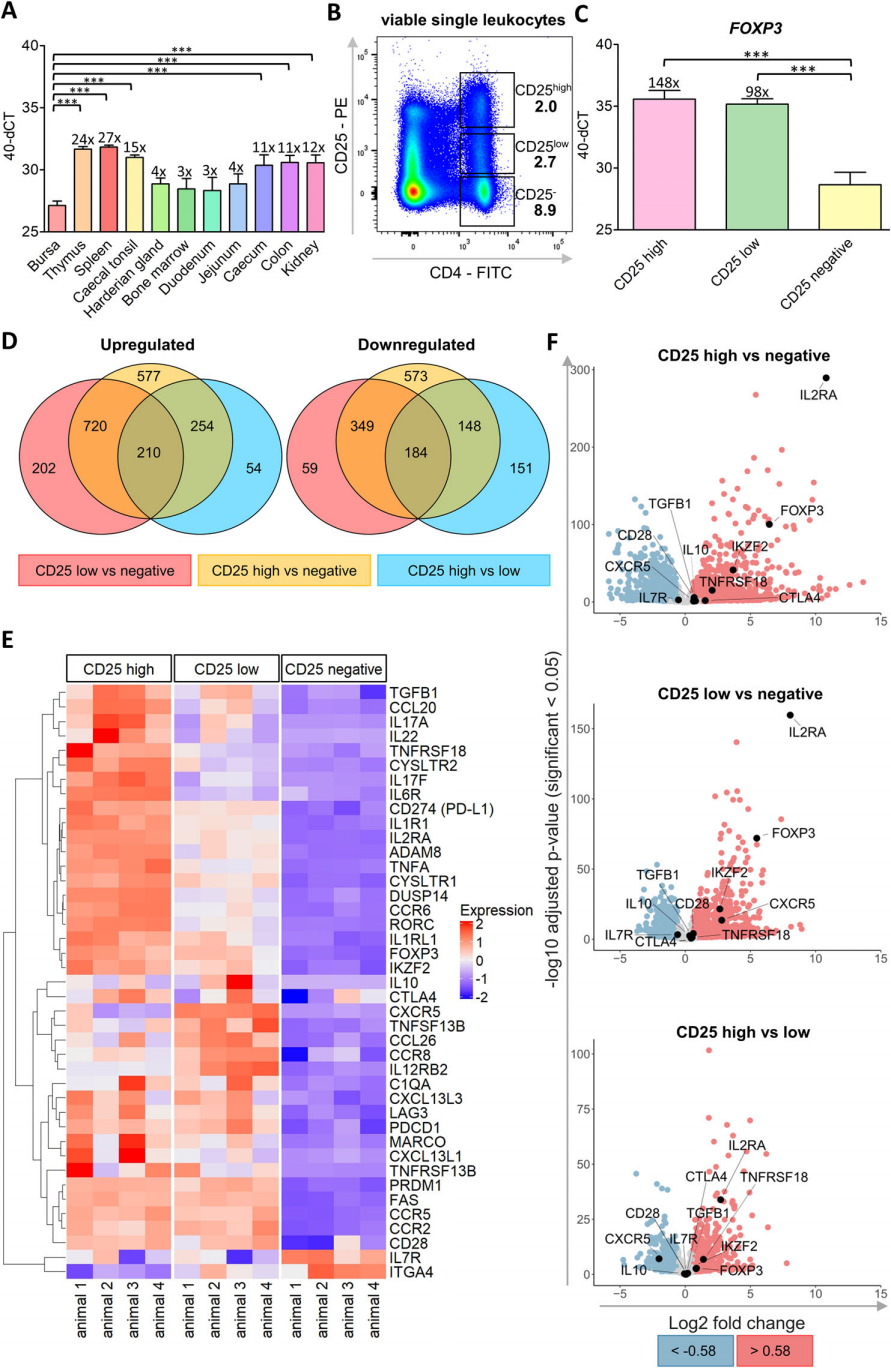
Treg‐associated genes are upregulated in both CD25^high^ and CD25^low^ CD4^+^ T cells, while both populations also express non‐Treg‐associated genes. (A, C) *FOXP3* expression levels across different organs measured by RT‐qPCR. Numbers above bars indicate mean fold change relative to the bursa (A), or CD25^negative^ cells (C). (B) Indicated subsets of splenic leukocytes were isolated by flow cytometry‐based cell sorting according to CD4 and CD25 expression, and (C) their *FOXP3* expression was quantified by RT‐qPCR. (D–F) Differential gene expression was analyzed by RNA sequencing of FACS‐purified CD25^high^, CD25^low^, or CD25^negative^ CD4^+^ T cells. (D) Venn diagrams with numbers indicating significantly up‐ or downregulated genes for each pairwise comparison. (E) Heatmap depicting gene expression of selected genes across conditions. Gene selection contains immune‐related genes among the top 100 DEGs ranked by fold change for each comparison and was complemented with genes with known positive or negative associations with Tregs (*TNFRSF18, CD28, PDCD1, CD274, IL10, TGFB1, IKZF2, FAS, LAG3, DUSP14, CCR8, CXCR5, IL7R, ITGA4*). Expression = Z‐score of normalized gene expression for each gene across conditions. (F) Volcano plots summarizing log_2_ fold changes (LFC) and *p*‐values for the same set of Treg‐associated genes for each comparison. (A, C) *p ≤ 0.05; **p ≤ 0.01; ***p ≤ 0.001; mean ± SD; one‐way ANOVA followed by Tukey's HSD post hoc test; (A) number of independent biological replicates (*n*) = 3; (B) representative of *n* = 3; (C) *n* = 4; (D–F) Negative binomial model; Wald test and likelihood ratio test, full model ∼ sex + CD25, reduced model ∼ sex; *n* = 4.

Because *FOXP3* is the lineage‐defining Treg transcription factor, we first quantified its expression in various lymphatic and non‐lymphatic chicken tissues using RT‐qPCR. We found the lowest *FOXP3* expression in the bursa, and the highest levels in spleen and thymus (27‐ and 24‐fold higher compared with bursa, respectively). Intermediate expression levels were observed in the caecal tonsil, caecum, colon, and kidney (11–15‐fold), whereas low expression was detected in the harderian gland, bone marrow, duodenum, and jejunum (3–4‐fold) (Figure 1A).

Given the high expression levels of *FOXP3* and the expectation that both nTregs and iTregs would be present in a secondary lymphoid tissue, we selected the spleen for further analysis. We found that chicken T helper cells vary in CD25 expression, and three distinct subpopulations can be identified by flow cytometry: CD4^+^/CD25^negative^, CD4^+^/CD25^low^, and CD4^+^/CD25^high^ (Figure 1B). Analysis of these subpopulations revealed a considerably higher *FOXP3* RNA abundance in CD25^high^ and CD25^low^ compared with CD25^negative^ cells (Figure 1C), thus confirming the co‐expression of *FOXP3* and CD25 in putative chicken Tregs.

To gain deeper insights into the transcriptional states and potential functional roles of CD25^high^, CD25^low^, and CD25^negative^ T helper cell subpopulations, we performed bulk RNA sequencing. We found 15,144 genes expressed in at least one sample and 5,018 differentially expressed genes (DEGs) across all groups. In individual comparisons (CD25^high^ vs. CD25^low^, CD25^high^ vs. CD25^negative^, and CD25^low^ vs. CD25^negative^), the most pronounced differences were found between CD25^high^ and CD25^negative^ cells, with 1758 significantly more and 1244 significantly less expressed genes in CD25^high^ cells (Table S2). We identified unique DEGs in each comparison, but the majority were shared across multiple contrasts (Figure 1D; gene lists provided in Table S5). However, neither GO nor Pathway analysis of these DEGs revealed any distinct groups that could be specifically related to Tregs. Therefore, we selected all immune‐related genes from the top 100 DEGs of each contrast and combined them (Table S5, highlighted in yellow) with a curated list of additional DEGs with known Treg importance in mammals (Table S1). The resulting heatmap (Figure 1E) demonstrates the highest abundance of many key genes for Treg function in CD25^high^ cells, including *FOXP3, IKZF2*, *CD274*, *TNFRSF18*, and *TGFB1*. Additional Treg‐associated genes like *CTLA4*, *IL10*, and *CD28* were expressed at similar levels in both CD25^high^ and CD25^low^ cells.

Interestingly, Treg‐associated chemokine receptor *CXCR5* was primarily expressed in CD25^low^ cells, and *IL7R* and *ITGA4* expression were highest among CD25^negative^ cells.

As expected, CD25 was differentially expressed in sorted subpopulations, aligning with protein expression. Importantly, *FOXP3* was significantly upregulated in CD25^high^ cells compared with CD25^negative^ (fold change 6.5; *p* = 5.41E‐101), as well as in CD25^low^ vs. CD25^negative^ cells (fold change 5.5; *p* = 1.12E‐72) (Figure 1F; Figure S1)). Along with Treg‐associated genes, several genes commonly associated with proinflammatory pathways, such as the Th17 cytokines *IL17A* and *IL17F* [16] and their master transcription factor *RORC* [17], as well as the Th1‐associated *TNFA* [18], were also identified as DEGs with high expression levels in CD25^high^ cells (Figure 1E).

### 2.2 Establishing an Anti‐Chicken CTLA‐4 Monoclonal Antibody

Identification through surface markers will be crucial for functional analysis of chicken Tregs. We identified *CTLA4*, a key molecule of mammalian Treg effector function, as highly expressed in both CD25^high^ and CD25^low^ compared with CD25^negative^ cells, but so far, no chicken‐specific antibody against CTLA‐4 has been formally described. However, the Immunological toolbox [19] provided us with four uncharacterized mAbs potentially targeting chCTLA‐4. We tested these mAbs on stably transfected human, rat, and chicken cell lines expressing chCTLA‐4 and found that while all four clones bound to chCTLA‐4 on HEK293 cells, only two mAbs (AV92 and AV94) were able to bind chCTLA‐4 on rat and chicken cells (Figure S2).

**FIGURE 2:**
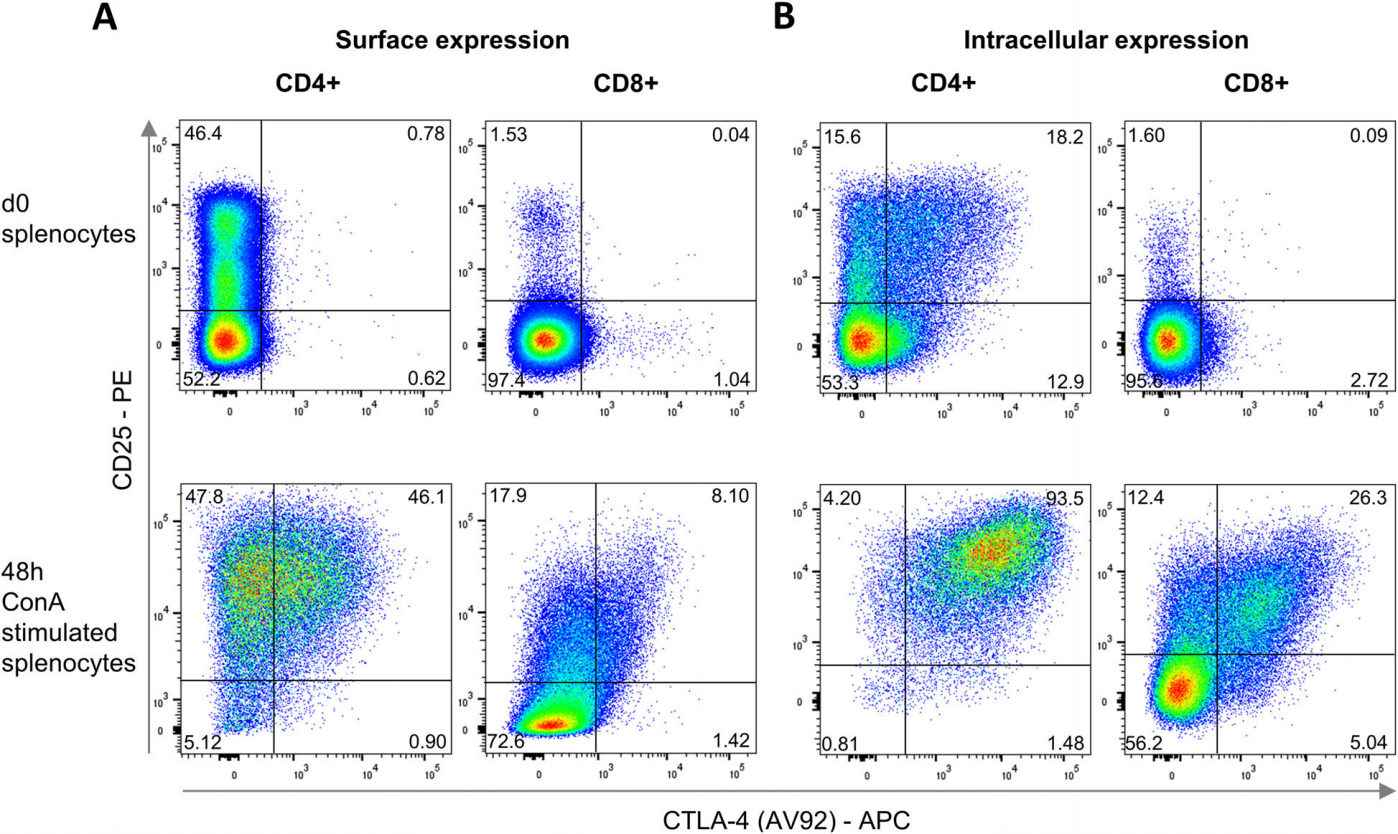
Activation‐induced upregulation of extra‐ and intracellular CTLA‐4 expression. (A) Surface and (B) ic CTLA‐4 expression detected by flow cytometry with mAb AV92 in freshly isolated (top) and 48 h ConA stimulated single viable CD4^+^ and CD8^+^ splenocytes. Gates were set based on isotype controls. Plots are representative of *n* = 3.

Flow cytometric surface staining with AV92 on CD4^+^ splenocytes did not detect CTLA‐4, and only a very small number of CD8^+^ cells were found to express this marker. However, ic staining detected CTLA‐4 in more than half of the CD25^+^ T helper cells. ConA stimulation resulted in increased CTLA‐4 surface detection on both CD4^+^ and CD8^+^ cells. Strikingly, while only a third of permeabilized ConA‐stimulated CD8^+^ cells were CTLA‐4 positive, CTLA‐4 was detectable in nearly all stimulated CD4^+^ cells (Figure 2).

### 2.3 Relation of Transcriptome and Protein Expression Levels

**FIGURE 3:**
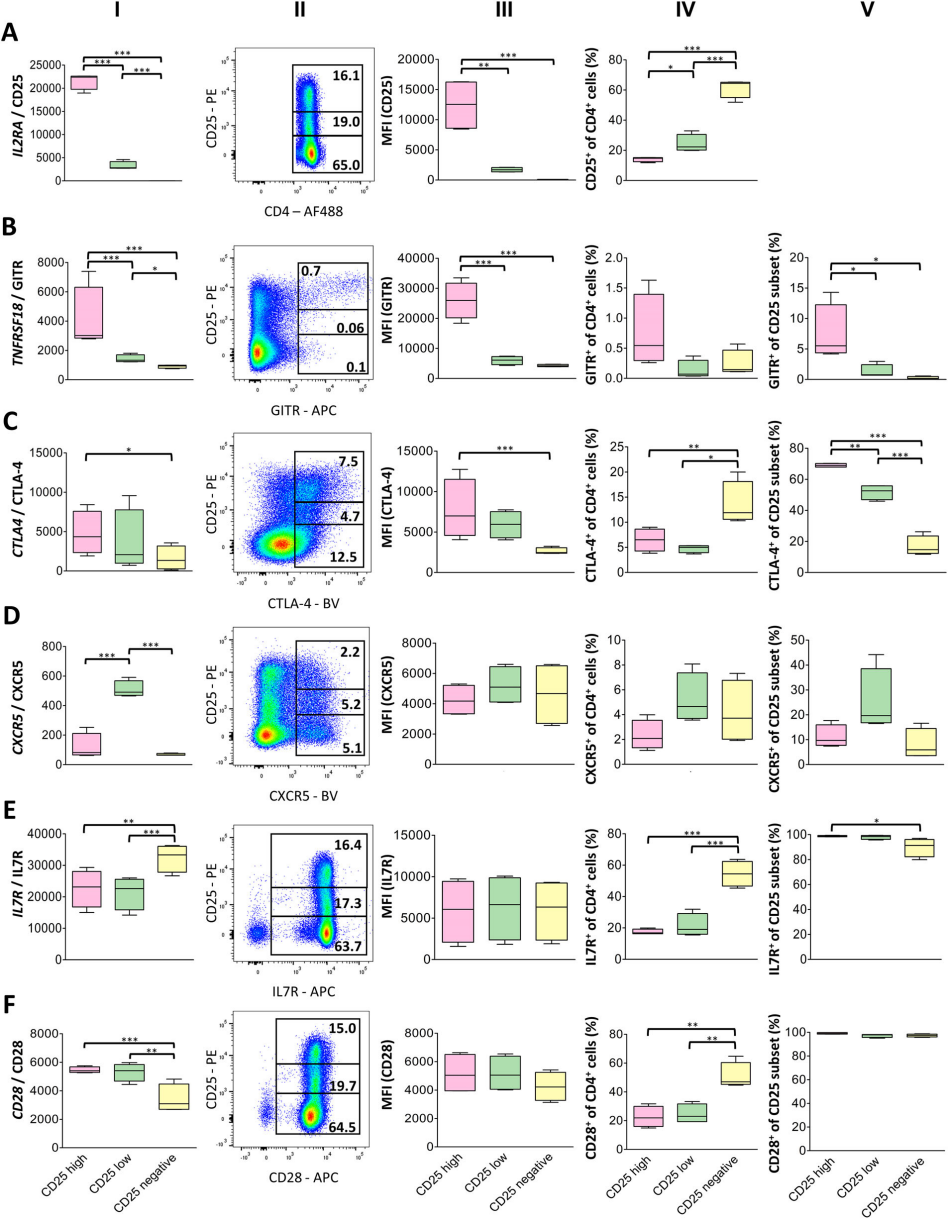
Comparison of RNA and protein expression of potential Treg markers reveals overall concordance. Expression of (A) *IL2RA* (CD25), (B) *TNFRSF18* (GITR), (C) *CTLA4*, (D) *CXCR5*, (E) *IL7R* and (F) *CD28* in CD25^high^, CD25^low^ or CD25 ^negative^ CD4^+^ T cells was quantified by RNA‐seq (column I) or flow cytometry (columns II–V). (Column II) representative dot plots gated for CD4^+^ viable single leukocytes; (column III) arithmetic mean of fluorescence intensity (MFI) of indicated markers in CD25‐based subsets (ic for CTLA‐4); (column IV) percentage of marker‐positive cells from total CD4^+^ T cells, and (column V) percentage of marker‐positive cells within the respective CD25 subset. *n* = 4, *p ≤ 0.05; **p ≤ 0.01; ***p ≤ 0.001; (column I) normalized gene expression values calculated using the median of ratios method implemented in DESeq2, mean ± SD; negative binomial model, Wald test; (column III–V) mean ± SD; one‐way ANOVA followed by Tukey's HSD post hoc test.

To identify potential Treg subpopulations, we correlated RNA and protein expression levels of all DEGs for which antibodies are available (Figure 3).

*IL2RA* RNA counts closely matched the mean fluorescence intensity (MFI) of anti‐CD25 staining, demonstrating identical CD25^high^, CD25^low^, and CD25^negative^ populations at both the RNA and protein levels (Figure 3A).

*TNFRSF18* (coding for GITR) showed decreasing RNA expression levels from CD25^high^ to CD25^negative^. This hierarchical pattern was mirrored by the MFI of GITR^+^ cells from the three different CD25‐based populations. However, only a small fraction of cells in each subset (6.1% of CD25^high^ to 0.1% of CD25^negative^) expressed GITR protein, resulting in a clearly defined GITR^+^ population among CD25^high^ cells (Figure 3B).

For CTLA‐4, RNA counts were compared with ic protein staining. RNA counts, MFI of positive cells, and the percentage of positive cells decreased from CD25^high^ to CD25^low^ cells. Notably, expression differences between CD25 subsets were much more pronounced at the protein than the RNA level (Figure 3C).

CXCR5^+^ subpopulations with a largely identical MFI were present in all three CD25 subsets. The highest percentage of CXCR5^+^ cells was observed in the CD25^low^ population, which was consistent with RNA abundance (Figure 3D).

IL7R was homogenously expressed on almost all CD25^+^ T helper cells. Interestingly, IL7R RNA abundance was significantly higher among CD25^negative^ cells, although flow cytometry identified an additional subset of CD25^negative^/IL7R^low^ and CD25^negative^/IL7R^negative^ cells (Figure 3E).

The flow cytometric staining profile for CD28 was very similar to IL7R, depicting a rather homogenous CD28 expression on almost all CD4^+^ cells and a minor subset of CD25^negative^/ CD28^negative^ cells. The slightly lower CD28 MFI in the CD25^negative^ subset was in line with the lowest CD28 RNA counts in these cells (Figure 3F).

In conclusion, RNA expression closely mirrored protein‐level expression for key Treg markers, yet the identity of specific Treg subpopulations, such as those marked by GITR, remained ambiguous.

### 2.4 T Helper Cell Heterogeneity Revealed by Single‐Cell Transcriptome Analysis

**FIGURE 4:**
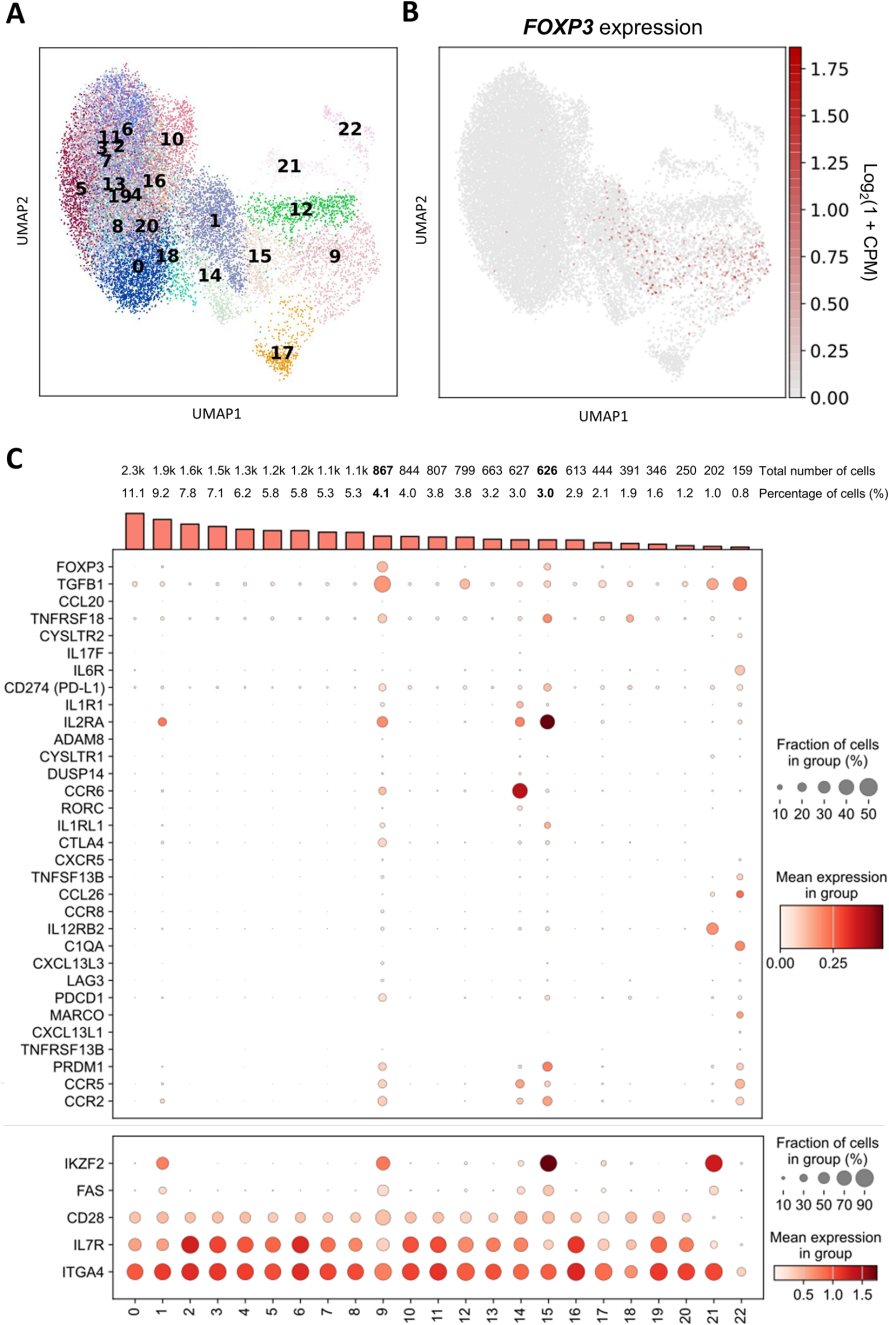
Single‐cell RNA‐seq analysis of CD4⁺ splenocytes reveals *FOXP3*‐expressing clusters. Unsupervised clustering of scRNA‐seq data identified 23 clusters, visualized (A) in a UMAP plot colored by cluster or (B) by *FOXP3* expression. (C) Dot plot showing expression patterns of immune‐related DEGs obtained by bulk sequencing and curated Treg marker genes (see Figure 1E). Genes with generally high expression levels are shown in a separate plot (lower panel) (C). (A–C) Clustering of batch‐corrected principal components using the Leiden algorithm with resolution set to 1.5; *n* = 4.

As bulk sequencing did not identify a specific Treg expression pattern in one of the CD25‐based subsets, we measured gene expression of flow cytometry‐sorted splenic CD4^+^ T helper cells by single‐cell RNA sequencing. For an unbiased identification of T helper subsets, integrated analysis of 20,838 cells from a total of four samples was performed. To enable optimal discrimination of *FOXP3*‐expressing cells, single‐cell RNA expression data were clustered at high resolution, resulting in 23 clusters (Figure 4A). Among these, cells in two clusters (9 and 15) exhibited significantly higher *FOXP3* expression than cells in other clusters (Figure 4B,C), while *FOXP3* expression in the remaining clusters was low and limited to fewer than 10% of cells per cluster.

The highest mean *FOXP3* expression was observed in cluster 9, which contained 4.1% of all cells and included 2192 marker gene candidates. Among the top five highly enriched marker genes (mean expression score, see Figure S3) was *SIVA1* (CD27BP), a protein that interacts with CD27, GITR, and Ox40, playing a crucial role in regulating Treg survival [20]. Cluster 15, representing 3.0% of all CD4^+^ splenocytes, exhibited intermediate mean *FOXP3* expression and had 290 marker gene candidates. *ITGB8*, a component of the integrin αvβ8 complex involved in TGF‐β activation in Tregs, was among the top three markers in this cluster [21] (Table S6.1 and S6.2).

Next, we considered expression patterns over all clusters of the intersection of the set of genes that were putative marker genes for either cluster 9 or 15, and the set of genes in Figure 1E (Figure 4C).

Both cluster 9 and 15 were characterized by significant over‐expression of *CTLA4, TNFRSF18, PRDM1* (Blimp‐1*), IL1RL1* (ST2, IL33R*), PDCD1*, *CD274* (PD‐L1), and *FAS. TGFB1* was particularly strongly expressed in cluster 9 (44.18% of cells in cluster 9 vs. 7.68% in all others, LFC = 1.93) and to a much lesser extent in cluster 15 (not significantly enriched). In contrast, compared with all other clusters, cluster 15 had by far the highest abundance of *IL2RA* (38.18%, LFC = 4) and *IKZF2* (82.27%, LFC = 4.95). In addition, both clusters revealed a reduced *IL7R* expression compared with most other clusters.

Although none of these markers were exclusively expressed or absent in the two *FOXP3* clusters, their combined presence resulted in distinct Treg signatures for clusters 9 and 15, highlighting the heterogeneity of putative Tregs.

### 2.5 Identification of Treg Subpopulations Based on CD25, GITR, and CTLA‐4 Expression

**FIGURE 5:**
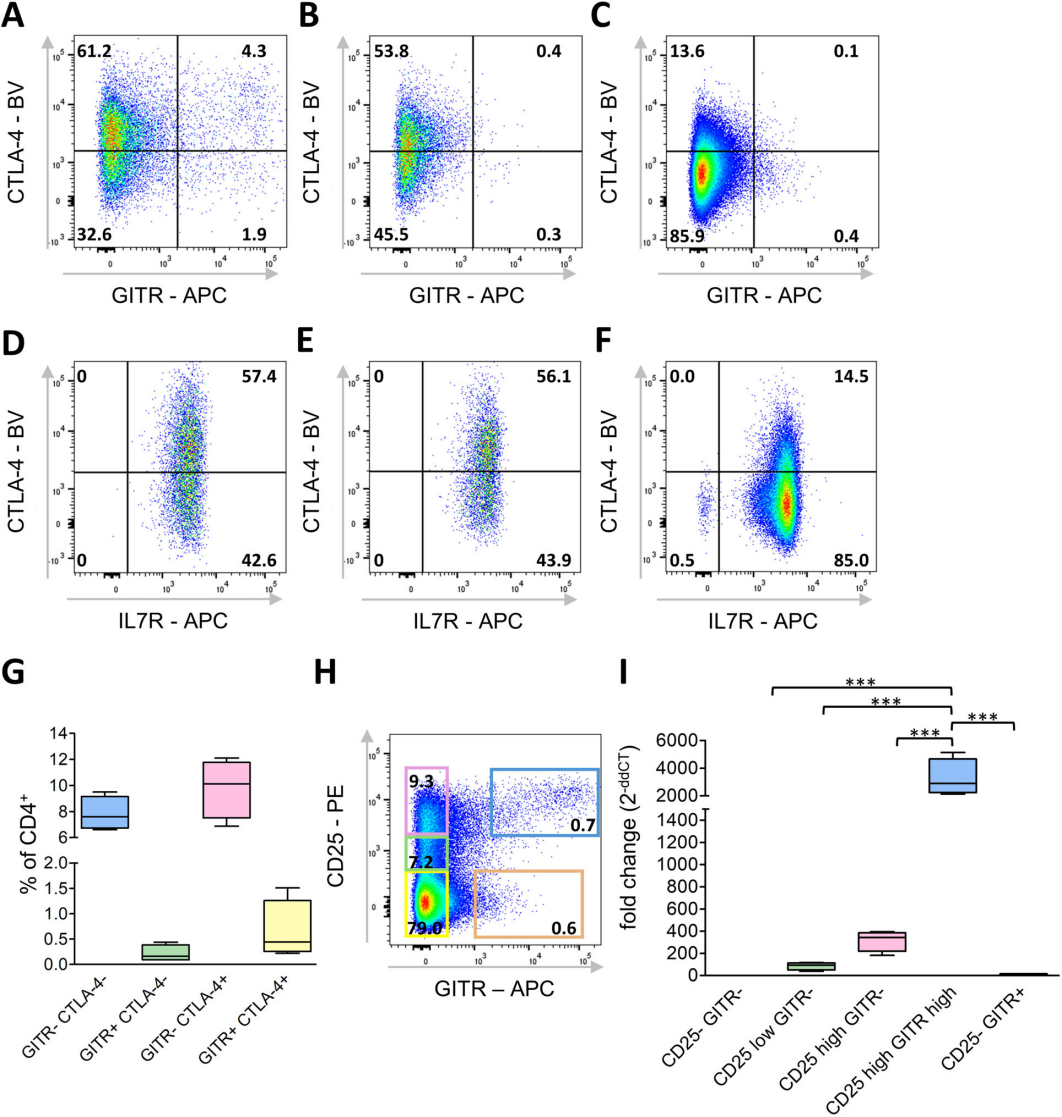
Identification of putative Treg subpopulations based on CD25, GITR, and CTLA‐4 expression. (A–G) Single viable CD4^+^ leukocytes from the spleen were analyzed by flow cytometry for GITR, ic CTLA4, and IL7R expression in (A, D) CD25^high^, (B, E) CD25^low^, and (C, F) CD25^negative^ subpopulations, or (G) in total CD4^+^ cells. Representative dot plots with the percentage of marker‐positive cells indicated. (H) Representative gating strategy for sorting of various CD25 vs. GITR subpopulations within CD4^+^ leukocytes and percentages of viable single CD4^+^ leukocytes indicated. (I) Relative *FOXP3* expression levels in the populations defined in (H), presented as fold change (2^−ddCT^) normalized to the CD25^−^ GITR^−^ subset. (G, I) Mean ± SD; (D–F) *n* = 2; (A–C and G–I) *n* = 4.

For several markers identified by scRNA‐seq in the *FOXP3* clusters, corresponding antibodies are available. Based on the scRNA‐expression profiles, we performed multicolor flow cytometry for CD4/CD25/CTLA‐4/IL7R and CD4/CD25/CTLA‐4/GITR. In contrast to expression differences on the RNA level, even in combination with ic CTLA‐4, all CD25^+^ T helper cells expressed high amounts of IL7R, and no IL7R^low^ population was detectable (Figure 5D,E). Therefore, identifying chicken Tregs as IL7R^low^ cells is not possible.

Analysis of CTLA‐4/GITR protein expression in CD4^+^ CD25^high/low/negative^ splenocytes revealed different subpopulations (Figure 5A–C,G), closely resembling the RNA data. We identified a CTLA‐4/GITR double‐positive population mainly in CD25^high^ cells, representing between 0.2% and 1.5% of total CD4^+^ cells. Additionally, a minor GITR^+^ CTLA‐4^−^ subset was present, accounting for less than 0.5% of CD4^+^ cells. According to scRNA‐seq data, these CD4^+^/CD25^high^/CTLA‐4^+^/GITR^+^ cells likely represent *FOXP3*‐expressing putative Treg cells.

Based on these data, we next determined whether CD25^high^/GITR^+^ expression can be used as a surface marker combination to identify chicken FoxP3‐expressing putative Tregs. Five CD4⁺ T cell subpopulations were sorted according to their CD25 and GITR expression profiles: CD25^−^/GITR^−^, CD25^low^/GITR^−^, CD25^high^⁄GITR^−^, CD25^high^⁄GITR^high^, and CD25^−^/GITR⁺ (Figure 5H). Subsequent RT‐qPCR analysis of *FOXP3* expression revealed that the CD25^high^⁄GITR^high^ subset displayed 2500–5100‐fold higher *FOXP3* mRNA levels relative to the CD25^−^⁄GITR^−^ population. By contrast, the CD25^high^⁄GITR^−^ subset exhibited considerably lower *FOXP3* expression (approximately 180–395‐fold relative to CD25^−^⁄GITR^−^). These data provide compelling evidence that the CD25^high^⁄GITR^high^ population corresponds to chicken cells characterized by high *FOXP3* expression, thereby establishing this surface marker combination as the first to precisely identify a subset of *FOXP3*⁺ putative chicken Tregs.

## 3 Discussion

The suppressive function of CD25^+^ chicken T helper cells and a potential contribution of CD25^+^ TGF‐β^+^ T helper cells to MDV and IBDV infection have been described by different groups [15, 22]. Additionally, we have recently identified the chicken orthologue of FoxP3, the master transcription factor of regulatory T cells [14]. However, these observations have not yet been integrated, and a detailed phenotypic description of chicken Tregs, which would enable their specific isolation, was lacking. Therefore, we have conducted detailed phenotyping of FoxP3^+^ T helper cells at various levels. As no cross‐reacting anti‐FoxP3 antibody is available [14] and because so far, all own attempts to generate an anti‐chicken FoxP3 antibody failed, we measured *FOXP3* expression based on RNA abundance.

We analyzed *FOXP3* expression from total tissue, by bulk sequencing of CD4^+^ CD25^negative^, CD4^+^ CD25^low^ and CD4^+^ CD25^high^ cells, and by single‐cell sequencing of splenic CD4^+^ cells. Both RT‐qPCR and bulk sequencing showed that *FOXP3* is present in CD25^+^ but not CD25^negative^ cells, with expression being slightly higher in CD25^high^ cells compared to CD25^low^ cells. Hence, putative Tregs are present among CD25^high^ and CD25^low^ cells. However, bulk sequencing already showed that Tregs can only be a fraction of CD25^+^ cells, because in addition to Treg markers, also many markers of other T helper cell subsets, like the Th17 transcription factor *RORC* (RORyC) [17], were expressed.

scRNA‐seq likewise confirmed the positive correlation between *FOXP3* and *IL2RA* expression, and one of the two Treg clusters displayed even the highest *IL2RA* abundance of all clusters. But a substantial abundance of *IL2RA* was also found in other clusters (Clusters 1 and 14), again demanding additional markers for Treg identification.

In both putative Treg clusters, many of the mammalian key Treg genes were significantly enriched: *CTLA4*, for contact inhibition [23, 24]; *PDCD1* (PD‐1) and *CD274* (PD‐L1), both usually upregulated upon Treg activation [25]; and *FAS*, important for Treg homeostasis [26]. We detected *PRDM1* (Blimp1), otherwise known as the regulator of plasma cell differentiation, but also shown to regulate growth and function of human Tregs [27]. Moreover, Blimp‐1 is a defining marker of mammalian effector Tregs (eTregs) and plays a critical role in their function. Hence, its presence in both chicken Treg clusters may indicate a similar role of Blimp‐1 in chickens and suggests the existence of chicken eTregs [28, 29]. We found *TNFRSF18* (GITR), which is constitutively expressed at high levels on human Tregs and supports Treg expansion and maturation [30]. In mammals, GITR is not exclusive to Tregs, and with cluster 18, we also identified a small CD25^negative^/GITR^+^ T helper subset in the chicken. Another cytokine receptor affecting mammalian Treg function is *IL1RL1* (IL33R), and IL‐33 binding to its receptor on Tregs can promote tissue repair [31]. In chickens, *IL1RL1* was among the top 10 significantly overexpressed genes in cluster 15, and was also expressed in the second Treg cluster 9.

In mammals, expression of the transcription factor *IKFZ2* (Helios) was initially thought to discriminate between induced and natural Tregs, but is now described as a marker of T cell activation for human, murine, and porcine T cells [32, 33]. This is in line with our observation that we find strong expression of *IKFZ2* in both *FOXP3* ^+^ clusters, with the highest *IKFZ2* and the highest *IL2RA* expression in *FOXP3* ^+^ cluster 15. Consistent with mammalian data, larger amounts of Helios were also detected in non‐*FOXP3* clusters, which probably contain many activated non‐Treg cells. The latter disqualifies Helios as a specific marker for chicken Tregs.

Besides the described similarities, there are also substantial differences between the *FOXP3* ^+^ clusters: Cluster 9 is characterized by high *TGFB1* and *CCR6* expression, whereas cluster 15 mostly lacks *CCR6* expression and expresses *IL2RA* more than any other cluster, arguing for distinct Treg subsets with different suppressive mechanisms. *TGFB1* may facilitate Treg induction and effector T cell suppression [34], and *CCR6* guides the cells toward inflamed tissue [35], whereas high *IL2RA* expression in Cluster 15 indicates suppression via IL‐2 sequestration [6]. This differs from earlier findings that showed the highest TGF‐β expression in CD25^high^ cells. This discrepancy may be due to the previous study examining membrane‐bound TGF‐β protein [15]. Another important suppressive mechanism of mammalian Tregs is IL‐10 secretion [36]. However, we did not detect *IL10* expression through single‐cell RNA‐seq analysis in any cluster and only minimal expression in bulk RNA‐seq data. The lack of this functional marker limits further functional differentiation of chicken Tregs. Nevertheless, our data demonstrate the heterogeneity of chicken Tregs, and perhaps sufficient and detectable IL‐10 induction would be possible using the right stimulation conditions.

To avoid ic FoxP3 staining, human Tregs are often addressed as CD4^+^/CD25^high^/IL7R^low^ cells [37]. Indeed, sc‐RNA‐seq data revealed relatively low *IL7R* RNA abundance in clusters 9 and 15. However, analysis of *IL7R* protein expression revealed homogenous surface staining, which does not allow for the discrimination of any CD25^+^/IL7R^low^ subset. This is a clear difference to humans and mice, which also demonstrates that RNA and protein abundance do not always correspond and emphasizes the necessity to evaluate each marker in the target species itself.

This also applies to CTLA‐4, which showed a comparably low but still quite specific scRNA‐expression in *FOXP3* ^+^ clusters. Together with the establishment of a chicken‐specific CTLA‐4 antibody, this made CTLA‐4 a promising candidate as a Treg marker. In mammalian Tregs, CTLA‐4 is located both on the surface and ic [38, 39, 40]. In contrast, while a small subset of CD8^+^ cells was CTLA‐4 surface positive, we found that in chicken T helper cells, CTLA‐4 was detectable almost exclusively intracellularly and in a much larger proportion of cells than expected based on the two *FOXP3* clusters. In mammals, ic CTLA‐4 is not restricted to Tregs but is also present in conventional CD4^+^ cells, where it is retained in Golgi vesicles under resting conditions [40]. Hence, the large fraction of chicken ic‐CTLA‐4^+^ cells is most likely comprised of different conventional T helper cell subsets. Consequently, our studies have shown that CTLA‐4 is poorly suited as a specific marker for freshly isolated chicken Tregs and potentially represents a more general activation marker. In addition, the requirement for ic staining prevents the isolation of viable cells. This might be different for in vitro experiments, as in vitro activation of CD4^+^ cells resulted in substantial CTLA‐4 surface expression.

Nevertheless, CTLA‐4 co‐staining was helpful to analyze the value of another promising candidate: *TNFRSF18* (encoding GITR), whose expression among CD25^+^ cells was restricted to *FOXP3* ^+^ clusters, and for which an antibody is available. Indeed, it was possible to identify a distinct CD25^high^/CTLA‐4^+^/GITR^+^ cell population, and in addition most GITR^+^ cells co‐expressed CTLA‐4. Importantly, our subsequent *FOXP3* expression analysis in sorted CD4⁺/CD25⁺/GITR⁺ cells revealed that this population is highly enriched for *FOXP3*‐expressing putative Tregs. As this population constitutes approximately 1% of T helper cells, it represents most likely only a subset of *FOXP3*‐expressing cells. But even though it is a small population, with CD4/CD25/GITR, for the first time, a combination of surface markers is now available that enables the isolation of living cells with Treg signatures and their subsequent functional analysis.

## 4 Data Limitations and Perspectives

The work would have benefited from the use of an antibody for chicken FoxP3, whose absence limited direct identification of FoxP3^+^ cells, necessitating reliance on indirect markers. No single marker combination fully captured the diversity of *FOXP3* ^+^ Tregs, highlighting their complexity and heterogeneity. This heterogeneity underscores the need to find additional markers to better capture *FOXP3* ^+^ populations.

While our data strongly suggest a transcriptional Treg signature, the lack of functional evidence leaves the immunosuppressive function of chicken FoxP3⁺ CD4⁺ T cells undetermined. This remains a limitation of the study. Furthermore, as our analysis was restricted to splenic cells and Tregs can exhibit tissue‐specific phenotypes, findings from other sites may differ. Nevertheless, our findings open new opportunities to specifically investigate the functional activities of cells with a Treg signature. Enhanced characterization of Tregs is crucial, especially given their role in infection processes. By providing precise tools for their identification, our study facilitates future research into immune regulation in chickens and the implications for avian health and disease.

## 5 Materials and Methods

### 5.1 Animals

Fertilized eggs from White Leghorn line M11 chickens (provided by Prof. Dr. Steffen Weigend, Federal Research Institute for Animal Health, Germany) were incubated and hatched at LMU Munich. Brown Leghorn chickens from the breeding colony of the Institute of Molecular Genetics for single‐cell RNA sequencing were housed at the Czech Academy of Sciences, Prague. All chickens were kept with ad libitum access to water and feed. One‐ to seven‐month‐old chickens of both sexes were euthanized for tissue collection.

### 5.2 Cell Lines

HEK293 and 2D8 [41] cells were cultured in RPMI medium (Thermo Fisher Scientific) supplemented with 10% FBS Advanced (Capricorn Scientific GmbH). Rat1 cells were additionally supplemented with 1% penicillin‐streptomycin (Merck KGaA).

### 5.3 Organ Collection and Preparation

Organs were collected in RPMI 1640 (Thermo Fisher Scientific) with 1% penicillin‐streptomycin (Merck KGaA) and homogenized through a stainless‐steel mesh to generate single‐cell suspensions. Leukocytes were enriched by density gradient centrifugation using Histopaque‐1077 (Merck KGaA), followed by washing with PBS (pH 7.2, Thermo Fisher Scientific).

### 5.4 Flow Cytometry

For all stainings, 1 × 10^6^ viable cells were incubated for 20 min on ice in the dark with primary antibodies in staining buffer (PBS pH 7.2, 0.1% bovine serum albumin, and 0.01% sodium azide (all Thermo Scientific)), followed by washing and 20 min incubation with fluochrome‐labeled isotype‐specific secondary antibodies.

Before ic staining, cells were fixed after surface staining and permeabilized using the Foxp3/transcription factor staining buffer set according to the manufacturer's instructions (eBioscience, Invitrogen). The gating strategy is shown in Figure S4.

Flow cytometric analysis was performed using a FACSCanto II with FACS DIVA software, and 100,000 viable CD4^+^ single cells were recorded per sample. Data were analyzed using FlowJo v10.10.0 software (all Becton Dickinson) (see Table S3 for antibody information).

### 5.5 Cell Sorting

For sort purification of cell populations for bulk RNA‐Seq and RT‐qPCR, cells were stained with CD4‐FITC/CD25‐PE (for bulk RNA‐Seq) and GITR‐APC (for RT‐qPCR), labeled with anti‐FITC MicroBeads, and CD4^+^ cells were magnetically separated on MidiMACS LS columns (both Miltenyi Biotec). Subsequently, CD4^+^/CD25 or CD4^+^/CD25/GITR subsets were sort‐purified on a FACSAria IIIu (Becton Dickinson) with purities >90%.

For single‐cell sequencing, CD4^+^ cells were sorted using a BD Influx (Becton Dickinson) without prior MACS separation.

### 5.6 RNA Isolation and Reverse Transcription

Cells were pelleted and stored at −80°C for RNA extraction. RNA was isolated using the ReliaPrep Cell RNA Isolation System or the SV Total RNA Isolation System (Promega). RNA concentration was measured with a Nanodrop ND‐1000 (Thermo Fisher) and a Quantus Fluorometer (Promega). Quality was assessed with a Bioanalyzer (Agilent), and only samples with a minimum RIN of 7.6 were used. For reverse transcription, 400 ng of RNA was processed with the GoScript Reverse Transcription Master Mix (Promega).

### 5.7 RT‐qPCR

Quantitative RT‐PCR (RT‐qPCR) was performed using optimized cDNA dilutiouns to ensure a linear amplification and optimal primer efficiency for each target gene.

5 µL cDNA was amplified in 25‐µL RT‐qPCR reactions as per manufacturer's instructions using GoTaq qPCR Master Mix (*RPL13*) or GoTaq Probe qPCR Master Mix (*FOXP3*) (both Promega). Primer and probe sequences and reaction conditions are listed in Table S4. For probe‐based assays, reactions included 100× CXR reference dye. RT‐qPCR was performed on a 7500 Real‐Time PCR System (Applied Biosystems). Expression was normalized to RPL13 (ΔCT). Relative gene expression fold changes were determined using normalized cycle threshold values (ΔCT), calculated relative to either the bursa or the CD25^negative^ population (2^^[−ΔCT(sample)−ΔCT(control)]^).

### 5.8 Bulk RNA Sequencing Analysis

RNA from sorted CD4⁺ subsets (CD25^negative^, CD25^low^, and CD25^high^) was used for bulk RNA sequencing. Strand‐specific 2 × 150 bp INVIEW transcriptome sequencing was performed (Eurofins Genomics, NGS Lab Constance), generating between 21 and 51 M read pairs per sample, with an average of approximately 36 M read pairs per sample. Quality control was conducted using FastQC v0.11.7 and MultiQC v1.17 [42, 43]. Alignment was performed using Salmon v0.14.1 [44] against the chicken transcriptome (bGalGal1.mat.broiler.GRCg7b) [45], with incompletely annotated chicken FOXP3 (GenBank‐ accession no. MT133687), BST2 (GenBank‐ accession no. MN326300), LAT (GenBank‐ accession no. BK061377), TNFA (GenBank‐ accession no. MF000729), CD19 (GenBank‐ accession no. OR836571), and EPO (GenBank‐ accession no. KR063574) cDNA sequences (provided by D.E.) appended to the transcriptome fasta file.

Subsequent analysis and graphical representation of the data were carried out in R v4.3.0 using the DESeq2 workflow, following the guidelines provided by the DESeq2 online workshop (GitHub—hbctraining/DGE_workshop) [46, 47].

### 5.9 Single Cell Sequencing Analysis

CD4⁺ splenocyte suspensions were processed using the Chromium Next Gem Single Cell 3' kit, v3.1, and the Chromium Next Gem chip G. Each sample targeted 5000 viable cells (271 to 321 cells/µL, 86%–95% viability). Sequencing was performed on the Illumina NextSeq 2000 platform using the P3 100 kit. To minimize batch‐specific effects, RNA from all four animals was processed in a single sequencing run.

For alignment, we used a modified chicken reference (bGalGal1.mat.broiler.GRCg7b) [45] with custom genes, as described in 5.8 (FOXP3, BST2, LAT, TNFA, CD19, EPO), filtered for protein‐coding genes, and indexed with CellRanger [48] (v4.0.11). Reads were aligned, and raw count matrices generated using “cellranger count”. Cell calling was performed with CellBender [49] (v0.3.0). Clustering analysis was conducted in Scanpy [50] (v1.9.3), with default parameters, unless otherwise noted. Cells were filtered (min_genes = 200, max_genes = 4000, max_counts = 15000, max 20% mitochondrial/ribosomal genes) and genes (min_cells = 3). PCA was performed on highly variable genes, selected from normalized log‐scaled counts, and batch effects corrected using harmonypy [51] (v0.0.9). Cells were clustered via the Leiden algorithm (resolution 1.5).

Treg clusters were identified by *FOXP3* expression. Additional markers were found by comparing the expression of each gene in all the cells of a given cluster with all the cells not in that cluster using a Wilcoxon rank‐sum test and a mean expression ratio score. For each gene, clusters were ordered by the mean expression of that gene. If the Treg cluster was not the top cluster for that gene, the score was 0. Otherwise, the score is the ratio of mean expression in the Treg cluster relative to the second‐highest cluster. GO analysis was performed via gProfiler [49], with human GO terms used for *FOXP3* and *IL2RA* due to a lack of chicken‐specific terms.

### 5.10 Transfection of 293, 2D8, and Rat1 Cells With chCTLA‐4‐Flag Plasmid

*CTLA4* was amplified from chicken cecal tonsil RNA by PCR with Q5 DNA polymerase, cloned into a pcDNA3.1/V5‐His expression vector [52] containing a Flag‐epitope by Gibson assembly, and transformed into NEB 5‐alpha Competent *E. coli* (all New England Biolabs). The sequence was verified by Sanger sequencing.

The CTLA‐4 expression plasmid was transfected into HEK293, 2D8, and Rat1 cells, using X‐tremeGENE 9 DNA Transfection Reagent (Merck KGaA), and stable transfectants were selected using G418 (Merck KGaA).

### 5.11 chCTLA‐4 mAb

We received four monoclonal antibodies (AV91, AV92, AV93, and AV94) from the UK Immunological Toolbox [19], proposed to target the chicken homolog of CD152 (chCTLA‐4). To validate their specificity, we performed flow cytometry on chCTLA‐4‐expressing HEK293, 2D8, and Rat1 cell lines, using nontransfectant cells as internal controls. Transfection efficiency was confirmed via Flag‐epitope staining (Figure S2).

### 5.12 Statistics

Statistical analysis was performed using R v4.3.0 or GraphPad Prism version 5.04 (GraphPad Software). Details about statistical tests are provided in the figure legends.

## Author Contributions

Isabell Naumann: Conceptualization, data curation, formal analysis, investigation, methodology, validation, visualization, writing – original draft, writing – review & editing; Edward S. Ricemeyer: Data curation, formal analysis, methodology, investigation, visualization; Daniel Elleder: Data curation, formal analysis, methodology, investigation; Jiri Plachy: Data curation, formal analysis, methodology, investigation; D. von La Roche: Investigation; Kim Vučinić: Data curation; Thomas W. Göbel: Supervision, methodology, resources; Bernd Kaspers: Conceptualization, formal analysis, funding acquisition, methodology, project administration, supervision, resources, validation, visualization, writing – review & editing; Simon P. Früh: Conceptualization, formal analysis, methodology, project administration, supervision, resources, validation, visualization, writing – original draft, writing – review & editing; Sonja Härtle: Conceptualization, formal analysis, funding acquisition, methodology, project administration, supervision, resources, validation, visualization, writing – original draft, writing – review & editing

## Ethics Statement

All animal work was conducted in accordance with European, federal, local, and institutional legislation and following the principles of the European Convention for the protection of vertebrate animals used for experimental and other scientific purposes and the German Animal Welfare Act.

## Conflicts of Interest

The authors declare no conflicts of interest.

## Supporting information




**Supporting File 1**: eji70106‐sup‐0001‐SuppMat.pdf.

**Supporting File 2**: eji70106‐sup‐0002‐TableS5.xlsx.

**Supporting File 3**: eji70106‐sup‐0003‐TableS6.1.xlsx.

**Supporting File 4**: eji70106‐sup‐0004‐TableS6.2.xlsx.

## Data Availability

All raw sequencing data from this study will be deposited in the European Nucleotide Archive (ENA) under project accession number PRJEB88203. In addition, the code for methods and clustering procedures will be released in the project's code repository https://github.com/esrice/chicken‐tregs.
