# Supplementary material for: Beyond FoxP3—Identification of a Chicken Regulatory T Cell Signature
Source: Eur J Immunol. 2025 Dec 19;55(12):e70106. doi: 10.1002/eji.70106 (PMC12716188; doi:10.1002/eji.70106)
Supplement: Supplementary file 1 — Supporting File 1: eji70106‐sup‐0001‐SuppMat.pdf. [file EJI-55-e70106-s001.pdf]

1. Supporting information

Supporting information Table 1: Markers used for Treg identification and phenotyping in conjunction with CD4 and CD25

**Gene**	**References**
FOXP3	Fontenot JD, Gavin MA, Rudensky AY. Foxp3 programs the development and function of CD4+CD25+ regulatory T cells. Nat Immunol. 2003;4(4):330-6. doi:{10.1038/ni904}<br><br>Le Bras S, Geha RS. IPEX and the role of Foxp3 in the development and function of human Tregs. J Clin Invest. 2006;116(6):1473-5. doi:{10.1172/jci28880}<br><br>Hori S, Nomura T, Sakaguchi S. Control of regulatory T cell development by the transcription factor Foxp3. Science. 2003;299(5609):1057-61. doi:{10.1126/science.1079490}<br><br>Khattri R, Cox T, Yasayko SA, Ramsdell F. An essential role for Scurfin in CD4+CD25+ T regulatory cells. Nat Immunol. 2003;4(4):337-42. doi:{10.1038/ni909}
CTLA4	Pacholczyk R, Ignatowicz H, Kraj P, Ignatowicz L. Origin and T cell receptor diversity of Foxp3+CD4+CD25+ T cells. Immunity. 2006;25(2):249-59. doi:{10.1016/j.immuni.2006.05.016}<br><br>Fontenot JD, Gavin MA, Rudensky AY. Foxp3 programs the development and function of CD4+CD25+ regulatory T cells. Nat Immunol. 2003;4(4):330-6. doi:{10.1038/ni904}<br><br>Hossen MM, Ma Y, Yin Z, Xia Y, Du J, Huang JY, et al. Current understanding of CTLA-4: from mechanism to autoimmune diseases. Front Immunol. 2023;14:1198365. doi:{10.3389/fimmu.2023.1198365}<br><br>Qureshi OS, Zheng Y, Nakamura K, Attridge K, Manzotti C, Schmidt EM, et al. Trans-endocytosis of CD80 and CD86: a molecular basis for the cell-extrinsic function of CTLA-4. Science. 2011;332(6029):600-3. doi:{10.1126/science.1202947}
TNFRSF18	Pacholczyk R, Ignatowicz H, Kraj P, Ignatowicz L. Origin and T cell receptor diversity of Foxp3+CD4+CD25+ T cells. Immunity. 2006;25(2):249-59. doi: {10.1016/j.immuni.2006.05.016}<br><br>Fontenot JD, Gavin MA, Rudensky AY. Foxp3 programs the development and function of CD4+CD25+ regulatory T cells. Nat Immunol. 2003;4(4):330-6. doi:{10.1038/ni904}

	Ronchetti S, Ricci E, Petrillo MG, Cari L, Migliorati G, Nocentini G, et al. Glucocorticoid-induced tumour necrosis factor receptor-related protein: a key marker of functional regulatory T cells. J Immunol Res. 2015;2015:171520. doi:{10.1155/2015/171520}
CD28	Guo F, Iclozan C, Suh WK, Anasetti C, Yu XZ. CD28 controls differentiation of regulatory T cells from naive CD4 T cells. J Immunol. 2008;181(4):2285-91. doi:{10.1155/2015/171520}
IKZF2	Kim HJ, Barnitz RA, Kreslavsky T, Brown FD, Moffett H, Lemieux ME, et al. Stable inhibitory activity of regulatory T cells requires the transcription factor Helios. Science. 2015;350(6258):334-9. doi:{10.1126/science.aad0616}
TGFB1	Levings MK, Bacchetta R, Schulz U, Roncarolo MG. The role of IL-10 and TGF-beta in the differentiation and effector function of T regulatory cells. Int Arch Allergy Immunol. 2002;129(4):263-76. doi:{10.1159/000067596}
IL10	Levings MK, Bacchetta R, Schulz U, Roncarolo MG. The role of IL-10 and TGF-beta in the differentiation and effector function of T regulatory cells. Int Arch Allergy Immunol. 2002;129(4):263-76. doi:{10.1159/000067596}
CXCR5	Kim YU, Kim BS, Lim H, Wetsel RA, Chung Y. Enforced Expression of CXCR5 Drives T Follicular Regulatory-Like Features in Foxp3(+) T Cells. Biomol Ther (Seoul). 2017;25(2):130-9. doi:{10.4062/biomolther.2016.075}
IL7R	Liu W, Putnam AL, Xu-Yu Z, Szot GL, Lee MR, Zhu S, et al. CD127 expression inversely correlates with FoxP3 and suppressive function of human CD4+ T reg cells. J Exp Med. 2006;203(7):1701-11. doi:{10.1084/jem.20060772}
PDCD1	Gianchecchi E, Fierabracci A. Inhibitory Receptors and Pathways of Lymphocytes: The Role of PD-1 in Treg Development and Their Involvement in Autoimmunity Onset and Cancer Progression. Front Immunol. 2018;9:2374. doi:{10.3389/fimmu.2018.02374}
ITGA4	Kleinewietfeld M, Starke M, Di Mitri D, Borsellino G, Battistini L, Rotzschke O, et al. CD49d provides access to "untouched" human Foxp3+ Treg free of contaminating effector cells. Blood. 2009;113(4):827-36. doi:{10.1182/blood-2008-04-150524}
CD274	Gianchecchi E, Fierabracci A. Inhibitory Receptors and Pathways of Lymphocytes: The Role of PD-1 in Treg Development and Their Involvement in Autoimmunity Onset and Cancer Progression. Front Immunol. 2018;9:2374. doi:{10.3389/fimmu.2018.02374}
FAS	Lim SP, Costantini B, Mian SA, Perez Abellan P, Gandhi S, Martinez Llordella M, et al. Treg sensitivity to FasL and relative IL-2 deprivation drive idiopathic aplastic anemia immune dysfunction. Blood. 2020;136(7):885-97. doi:{10.1182/blood.2019001347}

LAG3	Huang CT, Workman CJ, Flies D, Pan X, Marson AL, Zhou G, et al. Role of LAG-3 in regulatory T cells. Immunity. 2004;21(4):503-13. doi: {10.1016/j.immuni.2004.08.010}
CCR8	Whiteside SK, Grant FM, Gyori DS, Conti AG, Imianowski CJ, Kuo P, et al. CCR8 marks highly suppressive Treg cells within tumours but is dispensable for their accumulation and suppressive function. Immunology. 2021;163(4):512-20. doi:{10.1111/imm.13337}

Supporting information Table 2: Number of bulk RNASeq DEGs

	**CD25 negative vs. high**	**CD25 negative vs. low**	**CD25 low vs. high**
**Upregulated genes**	1761	1132	518
**Downregulated genes**	1254	592	483

Supporting information Table 3: Antibodies and staining conditions used throughout this study

Application	Antigen	mAb	Concentration / dilution
Cell sort/Flow cytometry	chCD4	2-6 (Dr. Sonja Härtle, LMU)	0.5 µg/ml
Flow cytometry	chCD4	(2-35) (Biorad)	2 ug/ml
Flow cytometry	chCD4	Fu11-2 (Olli Vainio, University of Turku, Finnland)	1:10
Cell sort/Flow cytometry	chCD25	28-4 (Prof. Thomas Göbel, LMU)	1:200
Flow cytometry	Flag	M2 (Merck)	0.5 µg/ml
Flow cytometry	chCD152	AV91 (Immunological Toolbox)	1:2
Flow cytometry	chCD152	AV92 (Immunological Toolbox)	1:10
Flow cytometry	chCD152	AV93 (Immunological Toolbox)	1:2
Flow cytometry	chCD152	AV94 (Immunological Toolbox)	1:4
Flow cytometry	chGITR	9C5 (1)	pure
Flow cytometry	chCXCR5	6A9 (2)	5 ug/ml
Flow cytometry	chCD28	2-4	pure
Flow cytometry	chIL7Ra	8F11E10 (3)	1.25 ug/ml
Flow cytometry	Viability marker eFluor506 (invitrogen)		1:1000

Flow cytometry	Viability marker eFluor780 (eBioscience)		1:1000
Flow cytometry	Isotype control IgG1	F71D7	5 µg/ml
Flow cytometry	Isotype control IgG2a		5 µg/ml
Flow cytometry	Isotype control IgM (BD)		5 µg/ml
Cell sort/Flow cytometry		Anti-mouse IgG1-FITC (Dianova)	1:200
Flow cytometry		Anti-mouse IgG2b-FITC (SBA)	1:200
Flow cytometry		Anti-mouse IgG2a AF488 (Jackson)	1:1000
Flow cytometry		Anti-mouse IgG3-AF647 (SouthernBiotech)	1:400
Flow cytometry		Anti-mouse IgG1 APC (Jackson)	1:1000
Flow cytometry		Anti-mouse IgG1 Brilliant Violett (Jackson)	1:200
Flow cytometry		Anti-mouse IgG2a AF647 (Invitrogen)	1:2000
Flow cytometry		Anti-mouse IgM FITC (SBA)	1:200
Flow cytometry		Anti-mouse IgM APC (SBA)	1:800
Cell sort/Flow cytometry		Anti-mouse IgG3-PE (SouthernBiotech)	1:200

1. Scherer S, Huhle D, Gobel TW. Identification of Chicken GITR and GITR Ligand, Proof of Their Mutual Interaction, and Analysis of Chicken GITR Tissue Distribution by a Novel Antibody That Reveals Expression on Activated T Cells and Erythrocytes. Immunohorizons. 2018;2(10):324-37. doi:{10.4049/immunohorizons.1800065}
2. Haertle S, Alzuheir I, Busalt F, Waters V, Kaiser P, Kaufer BB. Identification of the Receptor and Cellular Ortholog of the Marek's Disease Virus (MDV) CXC Chemokine. Front Microbiol. 2017;8:2543. doi:{10.3389/fmicb.2017.02543}
3. van Haarlem DA, van Kooten PJ, Rothwell L, Kaiser P, Vervelde L. Characterisation and expression analysis of the chicken interleukin-7 receptor alpha chain. Dev Comp Immunol. 2009;33(9):1018-26. doi:{10.1016/j.dci.2009.05.001}

Supporting information Table 4: Primers and PCR conditions used throughout this study

Gene	Appli-cation	NCBI accession no.	Forward Primer Sequence (500nM for qPCR)	Reverse Primer Sequence (500nM for qPCR)	Probe Sequence (300nM for qPCR)	T_A, Elongation	qPCR-Efficiency (%)
Foxp3	qPCR	MT133687	5'-AGTACGCCACAACCTGAGCCT-3'	5'-TTGGGGTCCTCTCAGCTCCGT-3'	5'-TGCGGGTGGAGAA-3'	60°C, 30 s	101.6
RPL13	qPCR	395849	5'-GAGGTGCCCGACTGTCAGAT-3'	5'-ATCGTCCGAGCAAACCTTTTGT-3'	/	59°C, 30 s	92.4
CTLA-4	Gibbson Assembly	424106	5'-GgacgatgacgataaggAAGTAATGGAAGTGACTCAGCCAG-3'	5'-gtgctggatatctgcagAGATATGTGGCAGTGTCTGG-3'	/	/	/

Supporting information Figure 1:

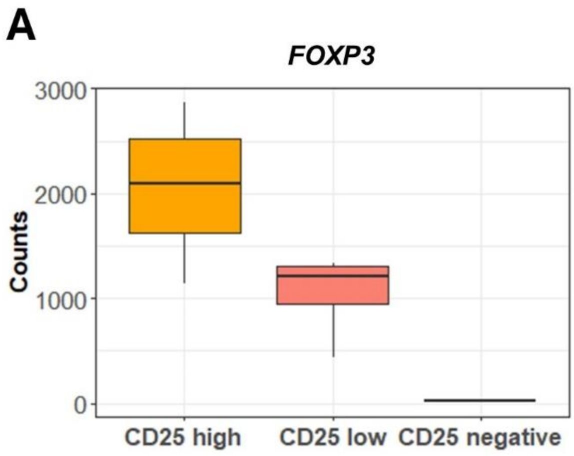

Normalized *FOXP3* expression in sorted CD4⁺ CD25 subpopulations analyzed by bulk RNA sequencing.

(**A**) Boxplot showing normalized *FOXP3* gene expression values, calculated using the median of ratios method implemented in DESeq2, in three $CD4^+$ CD25 subpopulations ($CD25^{high}$, $CD25^{low}$ and $CD25^{negative}$). n = 4.

Supporting information Figure 2:

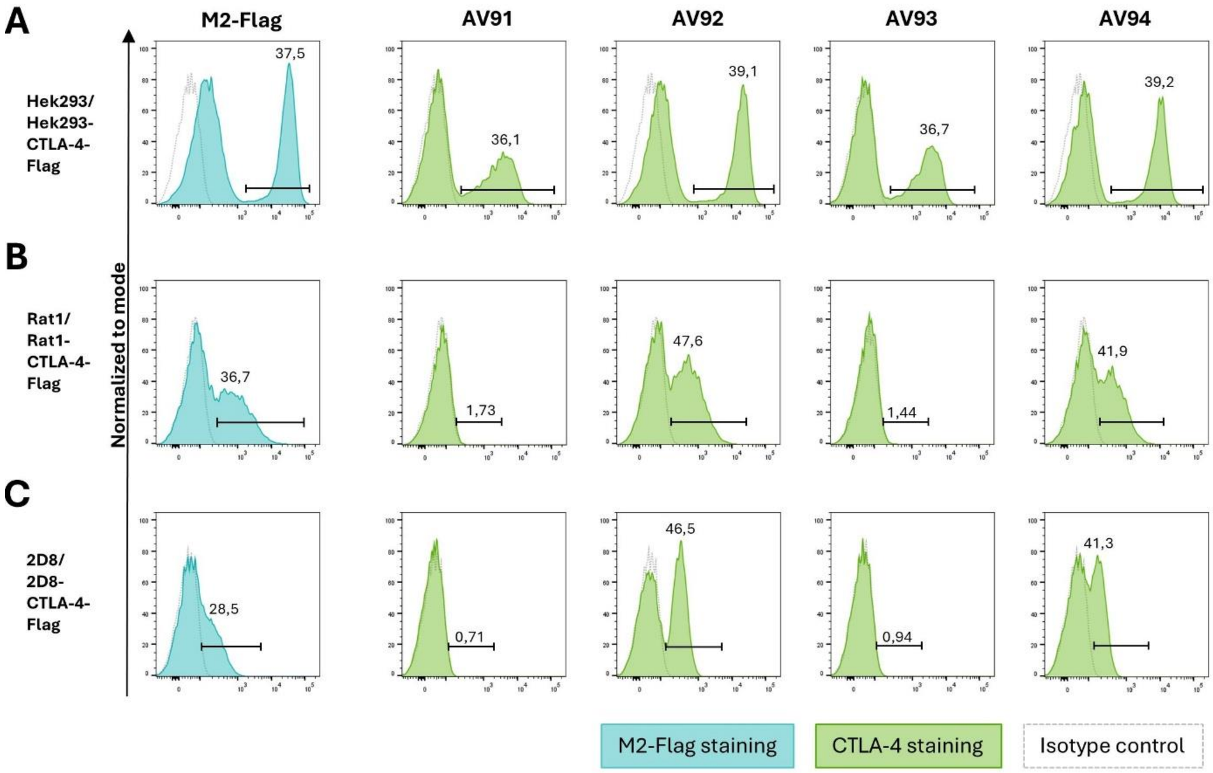

Staining of transfected CTLA-4–expressing cell lines with different CTLA-4 monoclonal antibodies and M2 anti-Flag mab reveals differential binding among the CTLA-4 mabs.

Representative flow cytometry surface staining patterns of M2-anti- Flag and anti-CTLA-4 mabs (AV91, AV92, AV93, AV94) on cell lines transfected with a pcDNA3.1 vector encoding chicken CTLA-4 with an N-terminal Flag-tag. (**A**) HEK293, (**B**) Rat1, and (**C**) the chicken derived cell line 2D8 (4).

4. Puehler F, Gobel T, Breyer U, Ohnemus A, Staeheli P, Kaspers B. A sensitive bioassay for chicken interleukin-18 based on the inducible release of preformed interferon-gamma. J Immunol Methods. 2003;274(1-2):229-32. doi:{10.1016/s0022-1759(02)00515-x}

Supporting information Figure 3:

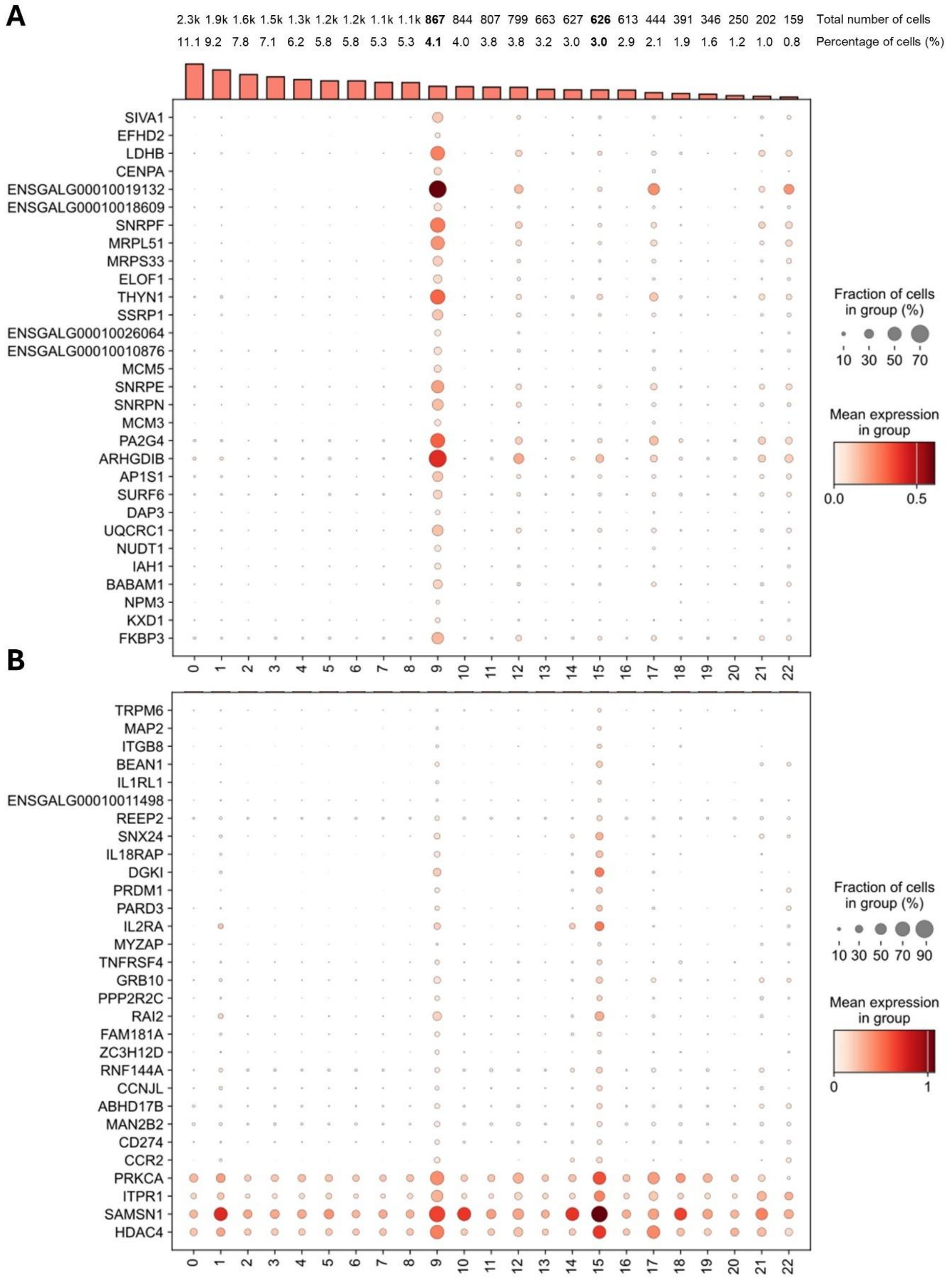

***FOXP3*-expressing clusters 9 and 15 display distinct transcriptional profiles.**
Dot plots showing the top 30 DEGs in cluster 9 (A) and cluster 15 (B), ranked by mean expression score within each respective cluster. Expression is displayed across all clusters. n=4.

Supporting information Figure 4:

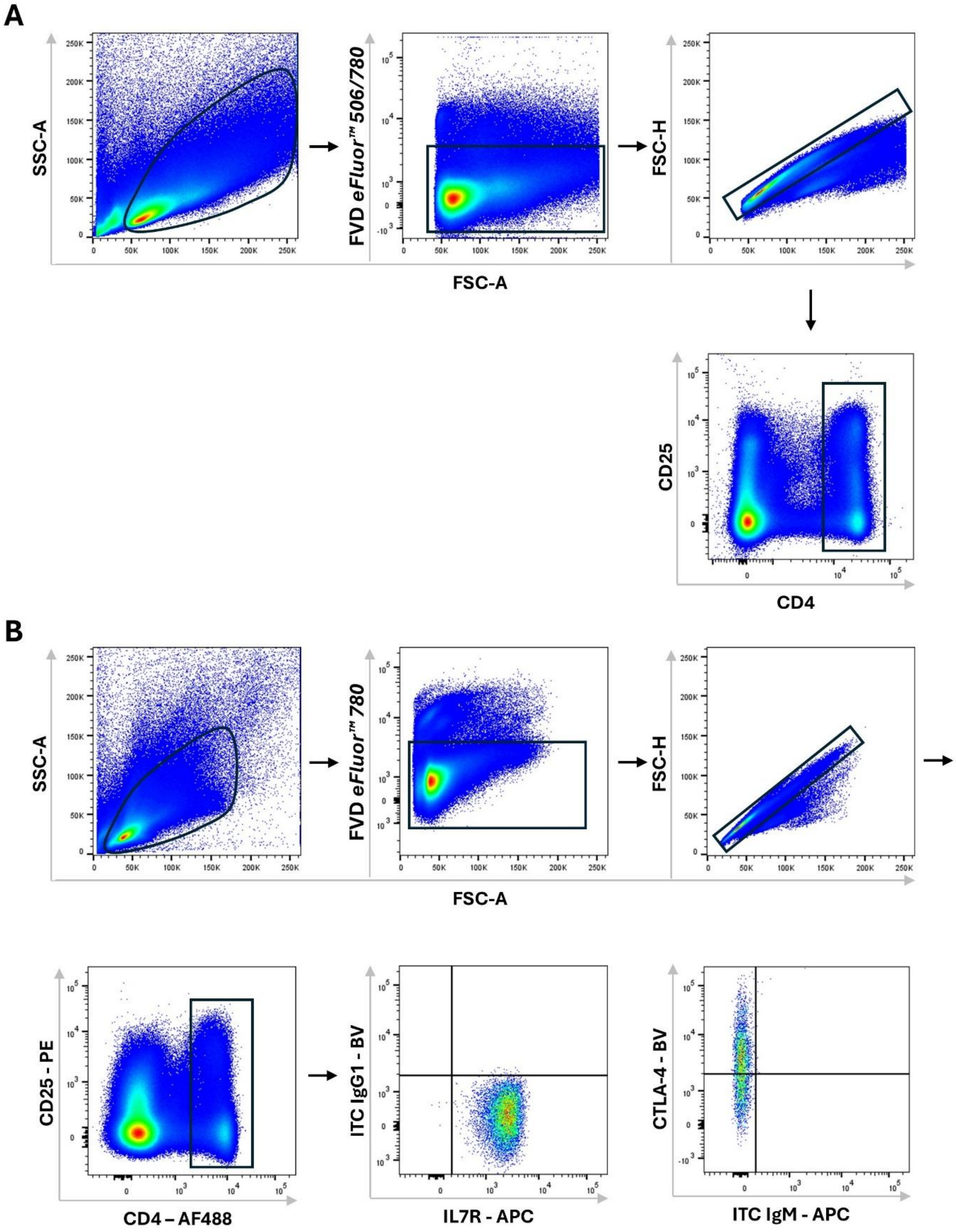

Flow cytometry gating strategy for CD4 T cells.

Surface staining (**A**) and intracellular staining (**B**) of splenocytes. Initial gating for leukocytes based on Forward and Side Scatter parameters was followed by exclusion of dead cells and gating for single cells. An additional gating step was applied to identify $CD4^+$ cells, with the final analysis focusing on $CD4^+$ single viable leukocytes. For these $CD4^+$ cells, isotype control stainings are displayed for IL7R (IgM) and CTLA-4 (IgG1).
